# A Revised Medical Nomenclature

**Published:** 1888-06

**Authors:** 


### *A REVISED MEDICAL NOMENCLATURE.*

Dr. J. W. S. Gouley has recently published a book, having for its objects: "First, to urge the official adoption of a stable basis of nomenclature and classification of the diseases of man; second, to place before the medical profession certain propositions directed to an improved classification of diseases; and, third, to awaken the attention of teachers to the necessity of ameliorating the nomenclature of medicine."

The profession will admit that great advantages would accrue from the adoption of a uniform system of nomenclature and classification, especially since vital statistics and the collective investigation of disease have of late years assumed so much importance. But that it is as yet possible to fix upon any stable basis of classification, we cannot believe, since pathology is still in an unsettled and speculative state. Many diseases are recognized only by symptoms, nothing of the anatomical changes being known, hence we cannot, as our author suggests, adopt an anatomical basis of nomenclature. The International Medical Congress might, however, adopt a uniform system of classification and nomenclature, subject to change in future years. For in all inductive sciences, hypotheses, theories, even systems and classifications must be purely arbitrary and subject to change with the discovery of each new truth. We believe, however, that there should be some censorial body to watch over the neologisms which are constantly being manufactured in our profession, a body like the French and Spanish academies, which might pass upon the accuracy and desirability of each newly coined word. There can be no body better suited for this purpose than a committee of the International Medical Congress.

If the new words proposed by Dr. Gouley were submitted to such a censorial body, few of them would survive the critical dissection to which they would be subjected. For example, *hypoæmia* is suggested as a substitute for *anæmia*, a word which means without blood, instead of deficient blood. But no Greek would have violated the rules of orthography and euphony by forming such a word as *hypoæmia*. *Hyphæmia* is the word desired. Of thirty-four words chosen to designate morbid conditions of the blood, at least twenty are improperly formed, the laws of Greek orthography and euphony being in each ease neglected.

The author also displays an unpardonable degree of carelessness in certain other words. *Nosonomy* is suggested as a word to designate the naming of diseases, a word which really means *the law of diseases*. *Nosonymy* is probably the word required.

Secondary affections are called *pathengenetic*, which our author informs us is derived from *pathos*, disease, and *eggenes*, born from, *i. e. ek*, *out*, and *genes*, born. But the word *eggenes* occurs in classical Greek literature, and means born in, from *en*, in, and *genes*, born. *Pathexegenetic* is a word which would mean, *born from the disease*. We are favored with an etymology of *ptomaine*, from *ptoma*, a carcass, and *in* or *en*, within, opposed to *ek*, without; when the termination *ine* is a purely arbitrary ending adopted by chemists to designate alkaloids and extractives. *Ine* is really derived from Latin *inus*, and means belonging to. We learn that leucocyte is derived from *leukos*, white, and *kustis*, a bladder, instead of *kutos*, a cell. On the whole, we have not for many years seen the language of Hippocrates tortured so unmercifully both from an etymological and typographical point of view. So careless have the printers and proof-readers been, that a correctly accented Greek word is quite an exception. Dr. Gouley's experiments in etymology only serve to prove one of the conclusions of his work: "That the nomenclature of disease can be much improved only after a very great change for the better shall be made in the fundamental science and associated arts of medicine," and, we might add, until more efficient classical scholars assume the task. Consequently, we will, for the present, continue to employ those old, "misused and improperly formed words," and will speak of *melancholy*, which has nothing to do with *black bile*; of *alopecia*, which has no relation to *foxes*, and of *arteritis*, which is not an inflammation of the *air tube*. But with the author's proposition to have the International Medical Congress adopt a uniform Latin nomenclature of disease, we heartily concur.

